# Evolutionary Breakpoints in the Gibbon Suggest Association between Cytosine Methylation and Karyotype Evolution

**DOI:** 10.1371/journal.pgen.1000538

**Published:** 2009-06-26

**Authors:** Lucia Carbone, R. Alan Harris, Gery M. Vessere, Alan R. Mootnick, Sean Humphray, Jane Rogers, Sung K. Kim, Jeffrey D. Wall, David Martin, Jerzy Jurka, Aleksandar Milosavljevic, Pieter J. de Jong

**Affiliations:** 1 Children's Hospital and Research Center Oakland, Oakland, California, United States of America; 2 Department of Molecular and Human Genetics, Baylor College of Medicine, Houston, Texas, United States of America; 3 Gibbon Conservation Center, Santa Clarita, California, United States of America; 4 Wellcome Trust Sanger Institute, Wellcome Trust Genome Campus, Cambridge, United Kingdom; 5 Institute for Human Genetics, University of California San Francisco, San Francisco, California, United States of America; 6 Genetic Information Research Institute, Mountain View, California, United States of America; University of Cambridge, United Kingdom

## Abstract

Gibbon species have accumulated an unusually high number of chromosomal changes since diverging from the common hominoid ancestor 15–18 million years ago. The cause of this increased rate of chromosomal rearrangements is not known, nor is it known if genome architecture has a role. To address this question, we analyzed sequences spanning 57 breaks of synteny between northern white-cheeked gibbons (*Nomascus l. leucogenys*) and humans. We find that the breakpoint regions are enriched in segmental duplications and repeats, with Alu elements being the most abundant. Alus located near the gibbon breakpoints (<150 bp) have a higher CpG content than other Alus. Bisulphite allelic sequencing reveals that these gibbon Alus have a lower average density of methylated cytosine that their human orthologues. The finding of higher CpG content and lower average CpG methylation suggests that the gibbon Alu elements are epigenetically distinct from their human orthologues. The association between undermethylation and chromosomal rearrangement in gibbons suggests a correlation between epigenetic state and structural genome variation in evolution.

## Author Summary

Mammalian genomes are remarkably stable (with few exceptions). In humans, wrong recombination events occur quite rarely, manifesting themselves in genomic disorders or cancer. On exceptional occasions, the rate of genome evolution has been accelerated by genome-wide reshuffling events giving rise to some highly derivative karyotypes. The genomes of gibbon species (Hylobatidae) are an example of accelerated genome structural evolution; gibbons display a rate of chromosome evolution 10–20 fold higher than the default rate found in mammals (one chromosome change every 4 million years). As we are interested in investigating the possible genetic causes of this phenomenon, we sequenced a considerable number of chromosomal breakpoints in the northern white-cheeked gibbon genome and analyzed the genomic features of these sites. We observe that the gibbon breakpoints are mostly associated with endogenous retrotransposons called Alus, which are normally abundant in the genomes of primates. Furthermore, our analysis revealed that gibbon Alus have a lower content of methylated CpG when compared to the orthologous human Alus. In mammals, CpG methylation is known to be responsible for keeping retrotransposons in a repressed state and protect genome integrity. We therefore suggest that a glitch in the methylation apparatus might have driven the higher genome recombination in gibbons.

## Introduction

Gibbons (Hylobatidae) are small arboreal apes that inhabit the tropical and semi-deciduous forests of Southeast Asia and a portion of South- and East-Asia; their closest relatives are the great apes (human, chimpanzee, gorilla and orangutan). They are an excellent model in which to study mechanisms of chromosomal rearrangement during evolution, because their chromosomes have been accumulating changes at an accelerated rate in comparison to other apes [1]–[3]. As a result of this instability, the four genera of the gibbon family possess four different karyotypes (2n from 38 to 52). The genome shuffling observed in gibbons is in striking contrast to the high degree of karyotype conservation found in the other hominoids: there is only a single inter-chromosomal rearrangement separating humans from the great apes [4], but more than 40 such rearrangements have taken place on the gibbon lineage. Recent estimates based on the inferred karyotype of the common gibbon ancestor suggest that the rate of chromosomal rearrangements in these species is 20 times higher than in other primates [3]. Given the great taxonomic diversity found within the family (four genera and fifteen species), it is tempting to speculate that segregating chromosomal changes mediated the speciation events in a relatively short time. The cause of this abundance of chromosomal changes is still undefined [5].

Primate genomes harbor millions of interspersed repetitive elements [6], creating numerous opportunities for Non-Allelic Homologous Recombination (NAHR) events to produce deletions, duplications and chromosomal rearrangements. Chromosomal rearrangements caused by NAHR are nevertheless quite rare, and even on an evolutionary time scale mammalian chromosomes have proven to be very stable. Comparison of multiple mammalian karyotypes indicates that the average rate of gross chromosomal rearrangements is only approximately two events over 10 million years [7]. Many repetitive DNA elements are rich in CpGs, which in mammalian cells are typically methylated. CpG methylation is an essential component of epigenetic mechanisms that maintain repetitive elements in a transcriptionally repressed state, thereby suppressing their proliferation [8],[9]. Cancer cells frequently exhibit a global decrease in genomic 5-methylcytosine, and it has been speculated that hypomethylation of repeat elements is an underlying factor in the high frequency of chromosomal rearrangements in cancer cells [10].

In search of an explanation for the abundance of evolutionary chromosomal changes in gibbons, we have now characterized the sequence and molecular structure of 57 breakpoint sites in the northern white-cheeked gibbon (*Nomascus leucogenys leucogenys*, NLE). We had previously created a high-resolution physical map of the break of synteny regions for this species [11], using the human genome as a reference. This map allowed us to localize the breakpoints within an 80 Kbp range. We have identified an association between the breakpoints and Alu retroelements, and we find that Alu elements in the gibbon are undermethylated in comparison to their human orthologues. Our findings suggest that epigenetic activity of Alu sequences may have facilitated karyotypic evolution and disruption of the uniform rate of chromosomal changes in gibbon species.

## Results

### Identification and sequencing of 57 gibbon breakpoints

To identify the breakpoints at the sequence level we selected 80 Bacterial Artificial Chromosome (BAC) from the *Nomascus leucogenys leucogenys* (NLE) genomic BAC library (CHORI-271) spanning the breakpoints of translocations and inversions. These BACs were selected from a high-resolution map that we constructed [11] and from a complementary list of gibbon BACs identified as spanning breakpoints by BAC End Sequencing (BES). Out of these 80 BACs, 23 were sequenced using a shotgun approach and assembled to high quality sequence (Table S1). The final assembled sequences were individually aligned by BLAT [12] to the most recent human genome assembly (hg18), and we identified the breakpoints between human and gibbon at the base pair level (Table S2). As we sequenced the BACs, we discovered multiple breakpoints inside the same clone in 8 of the 23 BACs including two cases previously reported by us. The complex structure of three of these BACs may be explained by their centromeric location in the gibbon (Table S2). In a few instances the presence of human segmental duplications did not allow for unambiguous mapping. To enrich our breakpoint dataset in a cost effective way, we pooled and shotgun sequenced at lower coverage the remaining 57 gibbon BACs (Protocol S1). This approach added 33 breakpoints to our dataset: 25 at the base pair level and 7 at the resolution of a small insert clone (about 6 Kbp) (Table S2). The remaining breakpoints could not be identified, due either to densely repeated regions or to lack of coverage. This brought the final number of breakpoints to 68 (57 at the base pair level). These results indicate that the frequency of breakpoints is higher than BES mapping alone can estimate. Hence assembly of the gibbon genome will be necessary to pinpoint all the breakpoints.

### Gibbon breakpoints show overlap with human- and gibbon-specific segmental duplications

**Figure 1:**
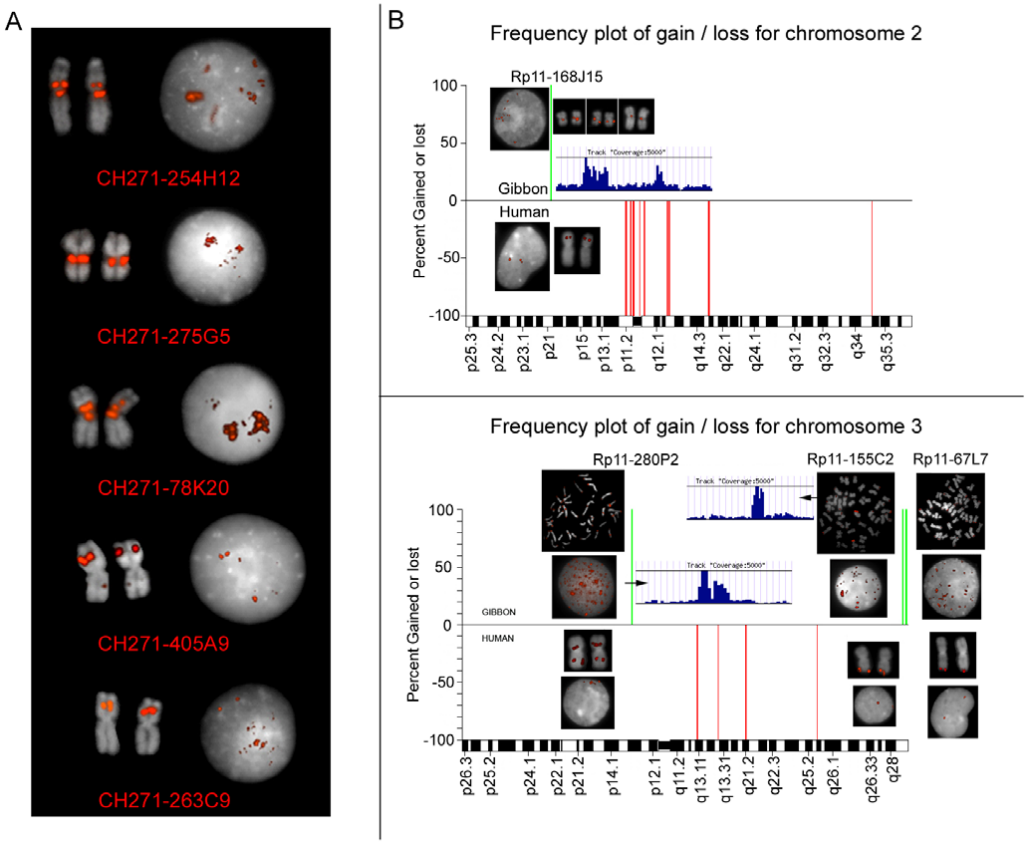
Analysis of gibbon specific segmental duplications. (A) Fluorescence *in situ* hybridization (FISH) experiments on NLE nuclei and metaphases using gibbon BACs spanning breakpoints which overlap with gSD. The fluorescent signals show a pattern typical of repeated sequences; (B) Images from the Array-CGH experiment using gibbon (test) versus human (reference) genomic DNA. Human chromosomes 2 and 3 are shown; duplications are represented in green and deletions in red. The duplications were validated by FISH on metaphases and nuclei of both human and gibbon using as probes the human BACs from the 32Kset. Duplicated regions present a higher depth of coverage of Trace Archives reads on the human genome as illustrated in the lateral panels.

In a previous study we uncovered a significant association between gibbon break of synteny regions, identified at a resolution of 80 Kbp, and human segmental duplications (hSD) [11]: 42% of the breakpoints were found to overlap with at least one hSD. In the current study we were able to identify the breakpoints at a higher resolution. This allowed us to further examine their relationship with SDs by measuring the correlation between a 1 Kbp window (−/+500 bp) including the breakpoint mapped on human and hSDs. We found that 15% of the breakpoints overlap with at least one hSD, which is significant (p = 0.0002) based on a random sampling simulation (performed as described in Materials and Methods) (Figure S1A). Recent studies have shown that a burst in duplication occurred in humans and chimpanzees after their divergence from other hominoids [13]–[15]. Thus we assume that the hSDs do not always correspond to gibbon SDs (gSD). As an assembled gibbon genome is not yet available, we used two methods to identify gSDs. First, we performed array-comparative genomic hybridization (array-CGH) of gibbon genomic DNA against human genomic DNA. This experiment allowed identification of large (>300 Kb) duplications/deletions that distinguish the two species. Second, following the method described by Bailey et al. [16], we mapped gibbon reads from the Trace Archives onto the human genome and identified putative gSD regions by detecting a higher depth of coverage by the reads (supporting online material). Of the gSDs identified by array-CGH, 37% were also identified as putative gSDs based on read coverage. Using the random sampling simulation approach mentioned above, we noticed that the overlap between the gibbon breakpoints and the gSDs is extremely large and statistically significant (Figure S1B), more than the overlap observed for the hSD. Examples of gibbon segmental duplications in breakpoints that could be detected by FISH are shown in Figure 1A. Even though the array-CGH and read-coverage-based gSD datasets do not show exact correspondence, we observed a significant correlation (p = 7.63e-9 by Fisher's Exact Test). We also validated, by Fluorescence in situ Hybridization (FISH), 11 duplications and 11 deletions identified by both methods (Figure 1B). Of note, the array-CGH results showed an excess of deletions in the gibbon relative to human (data not shown). We verified that 30% of the deletions are regions that are present in human at a higher copy number than in gibbon, confirming the occurrence of abundant human-specific duplication events.

### Gibbon breakpoints disrupt genes

We looked at the relationship between breakpoints and genes. When mapped onto the human genome, 53% (36 out of 68) of the breakpoints occur within a gene and 19% occur within non-coding transcripts (Table S2). We hypothesize that when a breakpoint disrupts a gene, the selective pressure on the sequence should be reduced as a consequence of loss of function, unless the truncated protein is rescued and still functional. As a measure of relaxed selective constraint on these disrupted genes, we calculated the dN/dS ratio between non-synonymous (dN) and synonymous (dS) substitutions between human and gibbon (using macaque as the outgroup). This analysis was carried out only on the 23 fully sequenced BACs (Protocol S2). The same method was applied to an equal number of randomly selected gibbon BACs sequenced by the NIH intramural sequencing center (NISC) comparative vertebrate sequencing project [9] (Table S3). This analysis showed a significant increase (p = 0.01, Mann-Whitney's U test) in the dN/dS ratio of gibbon genes when the breakpoint BACs are compared to the NISC BACs (Figure S2). It is worth noting that the p value becomes even smaller when the genes at <50 Kbp distance are considered, indicating a possible position effect. To confirm this trend, we sampled additional gibbon genes located at 500 Kbp and 1 M bp from the breakpoints, and found no differences when gibbon was compared to macaque (Table S4).

Frequently, genes affected by the breakpoints are part of clusters: the ABCC family on HSA 16, the ABCA family on HSA 17, the growth hormone cluster on HSA 17, *RFPL* on HSA 22, *MUC4* and *MUC20* on HSA3, *PLSCR* (phospholipid scramblase) on HSA 3. The association between breakpoints and gene-clusters has at least two biological implications. First, gene clusters result from duplication events that may cause genome instability through NAHR. Second, the presence of other genes with redundant functions could mitigate natural selection against chromosomal rearrangements that disrupt genes.

### Gibbon breakpoints are enriched in interspersed and simple repeats

**Figure 2:**
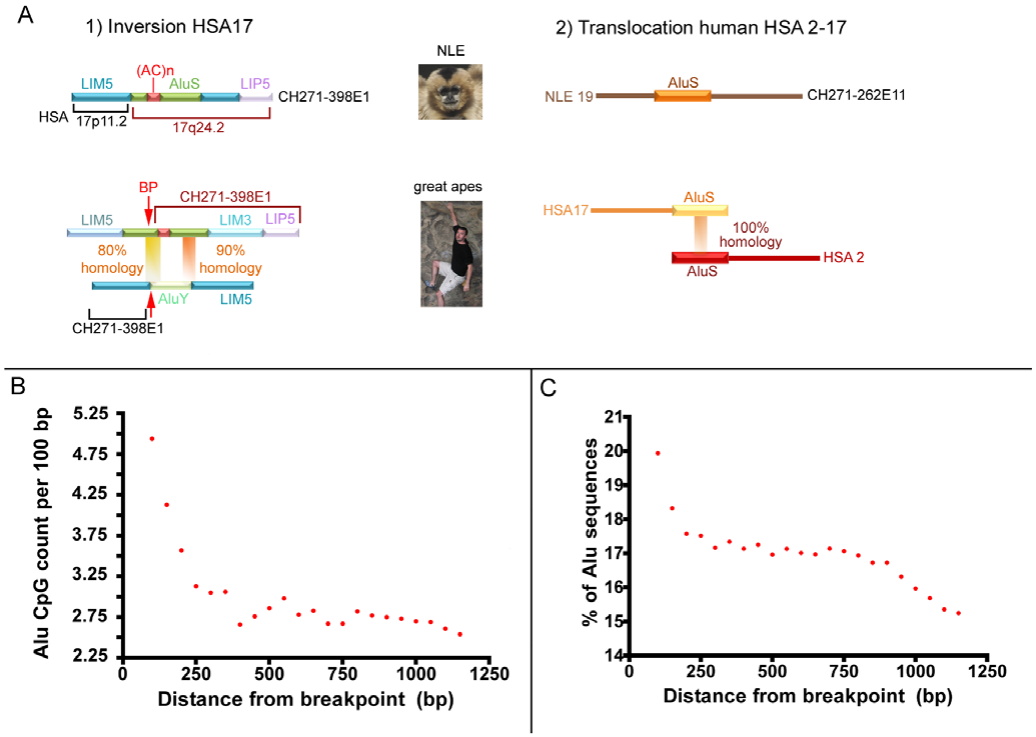
Examples of Alu–Alu mediated recombination events. (A) Two examples of Alu–Alu mediated recombination events in the gibbon discovered by comparing the gibbon and great apes orthologous locations. In the example 1 (clone CH271-389E1) the AluY and the AluS on human chromosome 17 (HSA17) share high homology in two locations. In gibbon the AluS was broken as result of the inversion and the AluY was lost. A simple scenario is illustrated in example 2: two identical Alus located at the breakpoint boundaries on human chromosomes 2 and 17 (HSA2 and HSA17) recombined and most likely caused the translocation whose breakpoints was identified in clone CH271-262E11; (B) distance from breakpoints to Alus showing a decline in Alu content when moving from the breakpoint. (C) the proportion of CpGs per 100 bp is higher for Alus or Alu fragments closer to the breakpoints.

The role of repeats in evolutionary or disease-causing chromosomal rearrangements is well documented [17]–[20]. We identified repeats within 150 bp of the 57 sequenced breakpoints with Repeat Masker. 81% of the breakpoints co-localized with at least one interspersed repeat. Alus and L1 LINEs are the most frequently represented, followed by simple repeats, as illustrated in Table 1. In 11 instances, one or more repeats span the breakpoint site in the gibbon. This could result either from an insertion after the breakage, or from a recombination event (Figure 2A). In the remaining cases, the repeats flank the breakpoint, and they are frequently truncated by the rearrangement event. Moreover, three breakpoints are next to blocks of repeats that were inserted sequentially in the gibbon genome, creating complex arrangements (Table S2).

**Table 1:** Detailed count of interspersed and simple repeats at the breakpoints.

Family	Repeat	Count	Total
**SINE**	Alu S	17	**27**
	Alu J	4	
	Alu Y	3	
	MIR	3	
**LINE**	L1	17	**20**
	L2	3	
**LTR**	LTR	8	**8**
**SIMPLE**	(AT)n	6	**11**
	(CA)n	2	
	Other simple	3	
**Other**	SVA	1	**5**
	HERVL	1	
	Charlie	1	
	Tigger 3b	1	
	HSMAR 2	1	

The regions that were analyzed for repeat content extended for 500 bp on each side of the breakpoint site. Only repeats at a distance <150 bp were counted and reported in this table and in Table S2.

Out of the 57 breakpoints, 11 co-localize with simple repeats of various types. Most of these breakpoints (6 out of 11) overlap with (AT)n-rich repeats which are either gibbon specific (CH271-254H12, CH271-171B20 and CH271-122E24) or shared by human (CH271-228C1, CH271-86M19, CH271-40A18). A different case is the breakpoint of a translocation HSA 3–5 that falls in the intra-genic tandemly repeated region (TR) of the mucin gene *MUC4* (3q29).

### Analysis of Alu CpG content and methylation

We were intrigued by the predominance of Alus at the breakpoint sites, as Alu-Alu recombination events have been reported as examples of Non Allelic Homologous Recombination (NAHR) [20],[21]. We verified that the proportion of Alus associated with breakpoints was significant when compared to other repeats by using a random sampling simulation (Figure S3A) (p = 0.001). At the same time this method showed that the association with LINE L1 in human was lower than expected by chance (Figure S3B).

We then looked for features of Alus that may be distinctive in gibbon compared to other hominoids. To carry out this analysis we used the 23 assembled BACs to represent portions of the gibbon genome surrounding the breakpoints. First, we observed a decline in Alu density within the BACs with increasing distance from the breakpoints (Figure 2B). Furthermore, Alu fragments at or near (<150 bp) the breakpoints were almost twice as CpG-rich as the remaining Alu sequences in the same BAC (4.5 CpGs/100 bp compared to 2.4 CpGs/100 bp; t-test p<0.001). As shown in Figure 2C, the number of CpG doublets per 100 bp of Alu sequence declines rapidly as the distance from the breakpoint increases. Active Alus contain a relatively high number of CpG dinucleotides, which are linked to active retrotransposition [9]. Normally, the epigenetic apparatus of the cell suppresses the activity of retrotransposons by adding methyl groups to cytosines in CpGs [22],[23]. Methyl-C tends to decay to T or A (therefore CpG become TpG/CpA) through a process known as CpG decay [22]. Our data thus suggest a higher concentration of “active” Alus associated with breakpoints.

**Figure 3:**
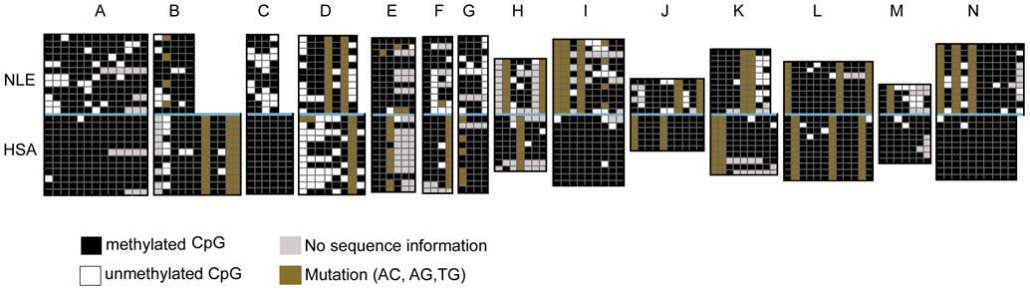
Results of bisulfite allelic sequencing of orthologous Alus in gibbon and human. Alus at orthologous locations in human and gibbon would have been inserted into the genome of the common ancestor and would therefore be the same age in the two lineages. Even though the Alus are the same age, there is a difference in the methylation levels at the CpG sites skewing towards lower methylation in the gibbon. One exception is the Alu D (CH271-263C9) which shows lower methylation in human.

We hypothesized that the higher rate of chromosomal breakage observed in gibbons is due to an active epigenetic state of these elements in the gibbon as compared to the common ancestor of the hominoids; the higher CpG content of these Alus suggests that they have been less methylated and consequently that they may have a different epigenetic state. The hypothesis predicts reduced CpG methylation of the gibbon breakpoint Alus in comparison to their human orthologues. We tested this prediction by performing bisulfite allelic sequencing of 14 orthologous Alus in human and gibbon, 8 of which were located near the breakpoint sites (<150 bp from the breakpoint) and 6 Alus outside of breakpoint regions but with similar CpG content to the breakpoint Alus (Materials and Methods and Table S5). As orthologous Alus are inserted in the genome of the common ancestor, we can safely assume that the CpG groups had the same amount of time to be methylated. Our results (Figure 3) demonstrate a significant reduction of CpG methylation in gibbon compared to human (p<0.001, Mann-Whitney U test).

## Discussion

**Figure 4:**
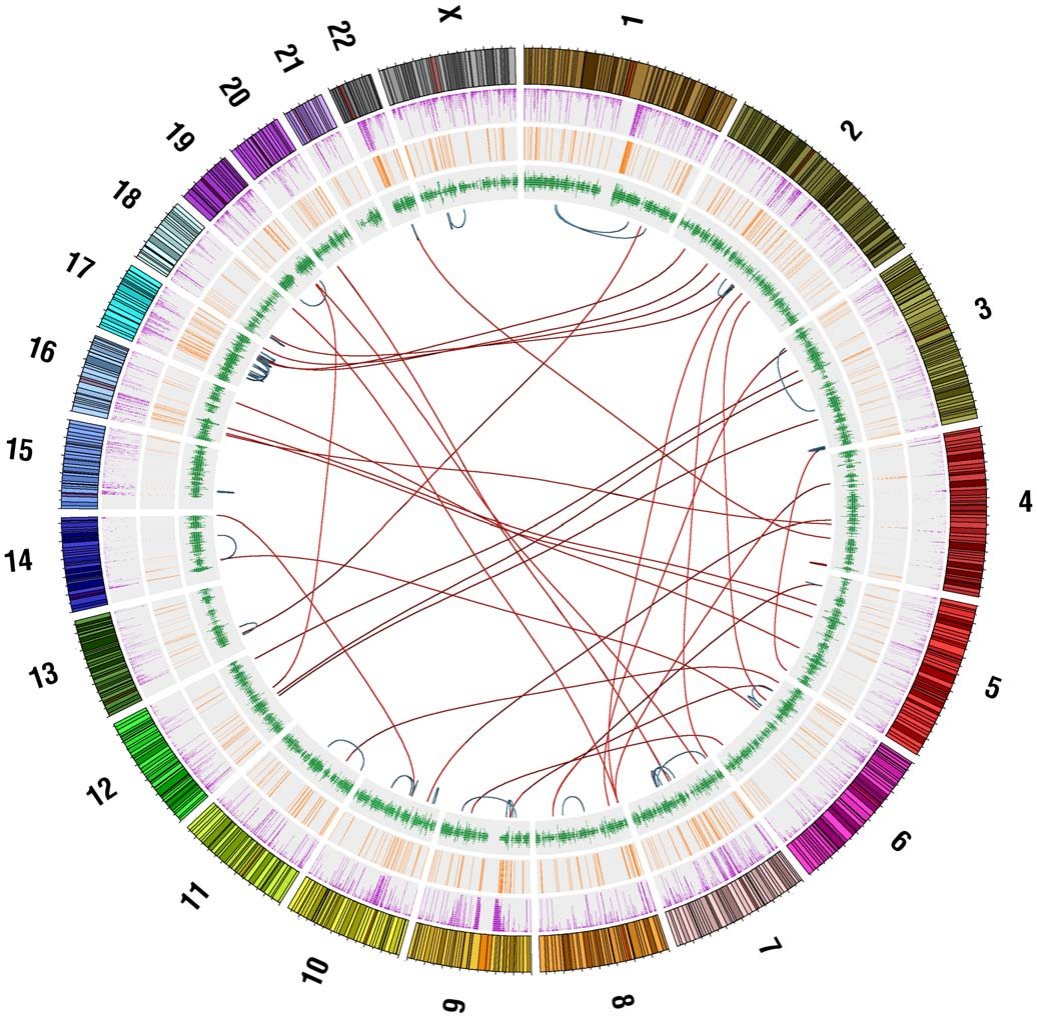
Visualization of gibbon rearrangements relative to the human genome. This visualization was generated using Circos software (http://mkweb.bcgsc.ca/circos/). The lines in the inner circle represent inter-chromosomal (red) and intra-chromosomal (blue) rearrangements in gibbon relative to human. The outer circles provide genomic context. The outermost circle displays human chromosomes along with genomic coordinates and G-banding stains (NCBI Build 36.1). Purple lines represent human segmental duplications from the UCSC Segmental Dups Track. (http://genome.ucsc.edu/cgi-bin/hgTrackUi?g=genomicSuperDups). Orange lines represent gibbon segmental duplications we predicted based on read coverage. Green lines represent human genes from the UCSC RefSeq Genes Track (http://genome.ucsc.edu/cgi-bin/hgTrackUi?g=refGene.

Gibbon species carry an extraordinary number of chromosomal rearrangements, accumulated in a relatively short evolutionary time (15–18 mya). In order to uncover a possible genetic source for the genomic reshuffling observed in these species, we carried on a detailed analysis of 57 sequenced synteny breakpoints between the northern white cheeked gibbon (NLE) and human. Our molecular analysis revealed a scenario which, at a first glance, is similar to that described in other primates [19], where segmental duplications and repeats play a major role in chromosomal rearrangements (Figure 4). But a broader analysis, which took into account epigenetic modifications, uncovered a possible explanation for the high frequency of evolutionary chromosomal changes. The gibbon breakpoints are associated with Alu elements that have an unusually high CpG content, and in the gibbon these Alu elements are less methylated than their human orthologues. This may indicate that the epigenetic state of these Alus has predisposed them to recombination.

In this study we were able to confirm the correlation between breakpoints and human SD which we had reported previously [11]. The higher resolution achieved in the present study, and the availability of gibbon sequences, allowed us to confirm association of the breakpoints with gibbon-specific SDs. As many breakpoints could not be mapped, due to the presence of these duplications (Table S2), the overlap is very likely to be more frequent than we have been able to demonstrate. It is noteworthy that we found only two breakpoints where SDs were present in both gibbon and human. As the intersection between gSD and hSD over the whole genome is much higher (32%), this observation suggests that the chromosomal rearrangements are mainly associated with “species-specific” duplications. The two cases of breakpoints in shared duplications may be explained by independent reuse of a breakpoint in regions susceptible to rearrangements [24]. Nevertheless, we do not believe that SDs can be considered an underlying cause of the breakpoints, as we have only few examples of erroneous recombination events in these regions (Table 2). Very similar observations have very recently been reported [5].

**Table 2:** Mechanisms of double-strand repair for gibbon rearrangements.

BAC	Rearrangement	Putative mechanism	Filling sequence	Micro-homology
CH271-372B11	t(HSA2;HSA9)	Alu-Alu recombination		
CH271-446I8	Inv(HSA7)	Alu-Alu recombination		
CH271-262E11	t(HSA17;HSA2)	Alu-Alu recombination		
CH271-398E1	Inv(HSA17)	Alu-Alu recombination		
CH271-383H22	Inv(HSA3)	Alu-Alu recombination		
CH271-350B17	Inv(HSA16)	NAHR (ABCC1-ABCC6)		
CH271-372B11	t(HSA9;HSA6)	NAHR of gSD		
CH271-286K22	Inv(HSA7)	NAHR of hSD		
CH271-261K6	Inv(HSA3)	NAHR of hSD (in human)		
CH271-261A22	Inv(HSA7)	NAHR of hSD (in human)		
CH271-261L1	Inv(HSA1)	NHEJ		AAGGTG
CH271-330D2	t(HSA16;HSA5)	NHEJ		CA
CH271-298N13	t(HSA8;HSA18)	NHEJ		TG
CH271-183B5	t(HSA8;HSA5)	NHEJ		GA
CH271-241J10	Inv(HSA1)	NHEJ	AAAAAAAAAATTTTCT	
CH271-78K20	t(HSA4;HSA16)	NHEJ	AATTCCAA	
CH271-171B20	Inv(HSA9)_1	NHEJ	ATACTACA(TA)_3_GA(TA)_5_TCCT	
CH271-86M19	t(HSA7;HSA20)	NHEJ	ATTCCAAGCCATATATTATTGG	
CH271-350B17	t(HSA4;HSA16)	NHEJ	CTCCAACCTT	
CH271-263C9	t(HSA22;HSA4)	NHEJ	GGGTTTCAGGG	
CH271-274L1	Inv(HSA17)_1	NHEJ	TGGTATGGAGCGAGCACCTCA	
CH271-449L10	t(HSA12;HSA19)	NHEJ	AAAA	
CH271-438C12	t(HSA10;HSA14)	NHEJ	AAC	
CH271-114O8	t(HSA5;HSA16)	NHEJ	ATGATG	
Traces 1744822164	Inv(HSA17)	NHEJ	GAAATAGAAATAAAAAC	
CH271-228C1	t(HSA7;HSA20)	Stem-Loop		

We were able to infer the mechanism for double strand repair on the bases of the molecular structure of the breakpoint for the 28 breakpoints that are listed in this table. The presence of long stretches of homology indicated that most likely NAHR recombination occurred while presence of micro-homologies or “filled in” sequences suggested NHEJ.

When studying evolutionary chromosomal rearrangement, it is tempting to search for sign of selection on genes that have been disrupted by the breakages. Recent work by Girirajan et al. [5] found evidence that 3 of their 11 genes disrupted by breakpoints exhibited signatures of relaxed evolutionary constraint (average dN/dS = 1.09). Our approach was different, as we looked at all the genes within the sequenced BACs, and compared them with randomly selected regions of the gibbon genome. We did, however, identify 5 genes in our sample that are disrupted by breakpoints and for which we had adequate coverage. Although we found that their values of dN/dS (average dN/dS = 0.56) were not as high as those reported by Girirajan et al. [5], subsequent analysis on the remaining dataset of all non-disrupted genes located within 50 Kbp of a break point, revealed a significantly reduced difference of dN/dS between gibbons and macaques (from p = 0.001 to p = 0.06) (Table S4). We hypothesize that some of these genes may still be functional, perhaps producing a smaller transcript, and that some may have become non-functional recently enough that non-synonymous substitutions have not had a chance to accumulate. Nevertheless, it appears that there are position effects on genes near to but not interrupted by breakpoints, perhaps due *cis* effects of chromatin in the breakpoint region, leading to changes in expression. A genome-wide expression assay would be needed to define the major trend for the genes that have been disrupted but this approach may be complicated by the scarcity of tissues available from this endangered species.

Breakage regions were found to co-localize with repeats. Whereas the known link between simple repeats and fragile genomic regions makes this observation intriguing, it is difficult to predict a cause-effect relationship between these repeats and the gibbon breakpoints. For many breakpoints we could readily observe that simple repeats were the result of gibbon-specific insertions by the repair mechanism after the break occurred. We therefore defined them as “filling” (Table 2) and we can assume that they followed the double-strand breaks. Our data point to a role for both Non-Allelic Homologous Recombination (NAHR) and non-homologous end joining (NHEJ) in double-strand break repair, with a prevalence of the latter. In 9 cases NAHR was driven by either Alu-Alu or SD mediated recombination (Table S2). In additional 15 cases, where long stretches of homology were not detected, we observed micro-homology or “filling” sequences which are both signs of NHEJ [25]. In NHEJ the double-strand breaks are fused together without a requirement for extensive homology. For the remaining breakpoints it was not possible to pinpoint a mechanism, even though the absence of homology would lead us to speculate that NHEJ or some other complex mechanism occurred in most of them [25].

While seeking a mechanism associated to the chromosomal reshuffling of gibbon species, our approach was to investigate Alu elements in more detail, given their higher concentration at the breakpoints. Independent evidence shows that this family of retrotransposons is particularly active in gibbons [26], strengthening our hypothesis. Our *in silico* and experimental data suggested that CpG cytosines in Alus are less methylated in gibbon than in human. CpG methylation has a major role in epigenetic suppression of endogenous retroelements in mammals. If this mechanism is attenuated, the repeated DNA sequences may threaten genome integrity: demethylation leading to an open chromatin structure at repeated sequences may cause structural and numerical variations [10]. Multiple examples of correlations between methylation state and genome structural variation have recently come to light in cancer cells, where disrupted methylation patterns are common [10],[27]. Furthermore, it was recently observed that hypomethylated blocks in tumor cell lines correspond to fragile regions of the genome and synteny breakpoints in the mouse [28]. This correlation suggests a common source of instability independent from genomic sequence and related to the epigenetic state of the DNA. O'Neill and colleagues showed that the genome of a hybrid between two species of Australian wallaby (marsupials) was hypomethylated when compared to the parental species [29]. In these hybrids a hypomethylated retroviral element was abnormally replicated causing an evident centromeric expansion. The same group also reported double-minute chromosome formation in mouse interspecific hybrids (*M. musculus×M. caroli*) [30]. Together with our findings this observation indicates that changes in methylation levels may explain perturbations of the uniform rate of genome evolution. Other mammal species (dog, mouse and rat), display very rearranged karyotypes and it will be important to investigate if the scenario we described in the gibbon is common to these species as well. Nevertheless, at the moment, the resolution of the synteny breakpoints for these species is still very far from the one needed to carry out an analysis comparable to the one we performed on the gibbon genome.

We have presented here a scenario that may explain the genome reshuffling observed in gibbon species: hypomethylation of certain Alu elements may predispose them to recombination. We are currently investigating the magnitude of the genome hypomethylation in gibbon repeat elements, and whether repeats other than Alu are involved. At the moment we can only speculate about the possible causes of the difference in levels of methylation of Alus that we observed in the gibbon. One hypothesis is linked to the observation that CpG methylation is disrupted in hybrids [29],[30]. Population genetics theories propose that speciation may occur after hybrid recombination, followed by inbreeding and reproductive isolation due to the new genetic make-up. This idea is well accepted for plants, and it has recently been proposed for gibbon species [31]. Hybridization may have gradually disturbed the apparatus responsible for the methylation of repeats in the hybrids, leading to higher numbers of chromosomal rearrangements [30]. Very recently the implications of a specific class of small RNAs (piRNAs) in methylation of repeats have been discovered. A rapid divergence of these sequences during speciation could therefore explain the reduction in the cytosine methylation efficiency in cross-species hybrids [32].

## Materials and Methods

### Random sampling simulation

The statistical analysis of the breakpoints repeat and duplication content was performed with the help of a C# application written in-house [11]. Tracks of genome-wide repeat content for different subcategories of repeats and for segmental duplications content were prepared for input to the simulation software using data from http://genome.ucsc.edu human genome (hg18 release). The measure we used counted up the existence of at least one element of the corresponding track in each region of the set and returned a detailed report for the set. To attain the simulation, the program reallocates randomly al the regions maintaining the chromosome of origin and size as the initial counterpart. The same measurements were taken for each random set after a reiteration of 5,000 times. The resulting sampling distribution was then plotted to compare the original set of regions with the global genomic landscape. The track relative to gibbon specific segmental duplication was built as result of our *in silico* analysis of the trace archives. Subsequently the latter tracks were used in order to perform different overlap measurements with the set of 57 breakpoints. When mapped on the human genome and the regions with ambiguous mapping are removed, the dataset corresponds to 120 regions of about 500 bp size (on average). Another set which we called “stringent set” was also used to determine the overlap with hSD. In this set all the breakpoints form two BACs (CH271-298N13 and CH271-372B11) known to be centromeric in the gibbon and containing multiple breakpoints, were excluded.

### In silico segmental duplication detection

Gibbon reads were downloaded from the NCBI Trace Archives and screened for quality. A total of 24,350,447 reads that passed quality screening were mapped to the human genome (build NCBI 36.1, UCSC hg18) using Pash [33],[34]. In order to remove highly ambiguous mappings, reads mapping to >500 locations with a score within 6% of its top mapping score were removed from consideration. Furthermore, reads that overlapped by >75% with repeats, as identified by RepeatMasker [35], were removed from consideration. A total of 15,518,707 mapped reads remained after filtering.

Putative gibbon segmental duplications were identified following the method outlined in Bailey, et al., 2002 [16]. The number of gibbon mapped reads was determined in 5000 bp windows across the human genome. The mean (31.11) and standard deviation (18.75) of mapped read counts was calculated across windows not overlapping with human segmental duplications. A read count cutoff of 3 standard deviations from the mean was applied meaning any 5000 bp region with >87 mapped reads was identified as a putative gibbon segmental duplication. This resulted in 1630 identified gibbon segmental duplications.

### Array CGH

32,855 BACs, spanning 95% of the human euchromatic genome, have been assembled and re-arrayed into 384-well microtiter dishes [36],[37]. DNA was purified, amplified using the DOP-PCR method, and spotted on CMT-GAPS coated glass slides (Corning, UltraGaps). Genomic DNA from NLE was obtained from blood and anonymous human reference DNA was obtained from Children's Hospital Oakland Research Institute. Labeling and hybridization were performed essentially as described by [38]. Hybridization images were generated by scanning the slides on a 4000B scanner (Axon). The images were first processed using GenePix Pro 5.1 (Axon Instruments). The primary experimental data (GenePix Results files) were subjected to fully standardized data-analysis (flagged spots removal, background subtraction and loess normalization) by uploading them to the BASE micro-array analysis software installation [39] which performs standard normalization. The final output was human chromosome specific plots of Log2ratio values vs chromosome location as well as a whole genome view.

### Fluorescence in situ hybridization (FISH)

Chromosome preparations were obtained from peripheral blood following standard procedures. Briefly, blood was incubated with cell culture media and phytohemagglutinin (GIBCO) for 72 hours (37°C, 5% CO2). Colcemid was then added (final concentration 0.05 ug/ml) and cells were harvested after a 1 hour incubation. Cells were spun down by centrifugation, the media was discarded and the pellet was resuspended in 8 ml of hypotonic solution. After incubating for 20 minutes, the standard fixative solution (1 part Acetic Acid, 3 parts Methanol) was added and cells were centrifuged at 2500 rpm for 5 minutes. The pellet was washed with fixative solution and cells were kept at 4°C overnight.

DNA from BACs was extracted using PureLink Miniprep kit (Invitrogen, Cat#K2100-10). FISH experiments were performed essentially as described by Lichter et al. [41]. BACs were labeled either with Cy3-dUTP or FITC-dUTP by standard nick-translation assay. Images were acquired using Nikon 80i microscope, equipped with CCD camera Cool Snap HQ2 (Photometrics) and software Nis Elements Br (NIKON). Elaboration of the images was done using Photoshop.

### Bisulfite allelic sequencing of Alu elements

Primers for 14 Alus (Table S5) were designed using “MethPrimer” (http://www.urogene.org/methprimer/) [42] making sure to target unique sequences flanking the Alu. Out of the 14 Alus, 8 were near the breakpoints (<150 bp); as our goal was to amplify Alus orthologous in human and gibbon, we had to take into account the synteny between human and gibbon and had to eliminate all the cases where the Alus were located across the breakpoint. The remaining Alus were located randomly in the gibbon genome but had to have a CpG content high enough to allow us to make a statistic.

The genomic DNA from whole-blood from gibbon and human was bisulfite converted using EpiTect Bisulfite kit (Qiagen, Cat.# 59104PCR) and the amplification was performed using the FastStart Taq DNA Polymerase (Roche, Cat#12032929001). PCR products were purified and cloned using TA-cloning procedures (Qiagen PCR cloning Kit, Cat.# 231124). We sequenced 12 clones for each Alu in order to have fair representation of all the alleles.

## Supporting Information

Figure S1 Random sampling simulations for human and gibbon segmental duplications. Random sampling simulations were carried on as described in Materials and Methods. Histograms were obtained for human SD (A) and the in silico set of gibbon SD (B). We also tested the overlap with a “stringent” sample (lighter color) where all the BP that in gibbon overlap with centromeres were removed. Even in this case it is evident that the overlap of the gibbon sample with both classes of SDs is significant.
(1.42 MB TIF)

Figure S2 dN/dS ratios for gibbon and macaque genes. The ratio of the average dN/dS compute for gibbons (vs. human) and macaques (vs. human) for all genes found within the fully sequenced BACs (Total), for genes found within the NISC database (NISC), for genes located within 50 kb (<50 kb) from the breakpoint found within the BAC sequences and genes located further than 50 kb (>50 kb) from the breakpoint found within the BAC sequences. p values were calculated using the nonparametric Mann-Whitney test.
(8.62 MB TIF)

Figure S3 Random sampling simulations for Alu and Line 1 elements. Random sampling simulations were carried on as described in Materials and Methods. The two charts show the histogram resulted from counting the overlap between random regions of the human genome and Alu (A) and Line (B). The random sampling was repeated 5,000 times in both cases. The corresponding value for the gibbon dataset is indicated by the blue arrow.
(1.51 MB TIF)

Table S1 Sequenced Gibbon BACs. The fully sequenced and assembled 23 gibbon BACs from the genomic BAC library CHORI-271 (http://bacpac.chori.org/library.php?id=228) are reported here with the corresponding accession numbers.
(0.04 MB DOC)

Table S2 Gibbon breakpoints mapping information and annotations. We report here the sequencing status and annotations for 80 BACs used in this study. All the BACs were End-sequenced and mapped on the human assembly (hg18). The breakpoint sequence was obtained for 46 BACs whereas for 7 clones we identify Trace Archives mate pairs whose mapping indicated the presence of the breakpoint but no breakpoint sequence was found. For 24 clones the breakpoints could not be narrowed down at a resolution higher than a BAC clone. For each breakpoint sequence repeat, segmental duplications and gene contents are annotated. Abbreviations: gSD = gibbon segmental duplication, hSD = human segmental duplication.
(0.11 MB PDF)

Table S3 Estimates of dN/dS for each gene within the BAC sequences. The tables report the estimates of dN/dS for genes within the NISC BACs (left-most table) and for the fully sequenced gibbon BACs (center table). The small table on the right illustrates the averaged estimates of dN/dS for genes where human, gibbon and macaque sequences were available. (*) Hypothetical genes were removed to provide a more stringent analysis and genes found within BAC CH271-262e11 were omitted because they belong to a gene cluster family with high sequence identity. This sequence identity along with their position upstream and downstream of the BP leads to uncertainty in alignment data and gene coordinates.
(0.04 MB XLS)

Table S4 Estimates of dN/dS for genes located within specified distances from the breakpoint tested in the study. Estimates of dN/dS for each gene found within 400–600 kb from the BP where human, gibbon and macaque sequences were available (left-most table). Estimates of dN/dS for each gene found within 0.9–1.1 Mb from the BP where human, gibbon, and macaque sequences were available (center table). The small table on the right side summarizes the average dN/dS values at various distances from the BP tested in the study. Distances were determined by calculating the minimum distance from either the start or end of the gene to the BP.
(0.03 MB XLS)

Table S5 Primers used for Bisulfite Allelic sequencing. The table lists the primers that have been used to amplify the 14 Alus and carry on allelic bisulfite sequencing.
(0.03 MB XLS)

Protocol S1 Breakpoints mapping strategy.
(0.03 MB DOC)

Protocol S2 dN/dS analysis.
(0.03 MB DOC)
