# Supplementary figures and images for: Evolutionary Breakpoints in the Gibbon Suggest Association between Cytosine Methylation and Karyotype Evolution
Source: PLoS Genet. 2009 Jun 26;5(6):e1000538. doi: 10.1371/journal.pgen.1000538 (PMC2695003; doi:10.1371/journal.pgen.1000538)

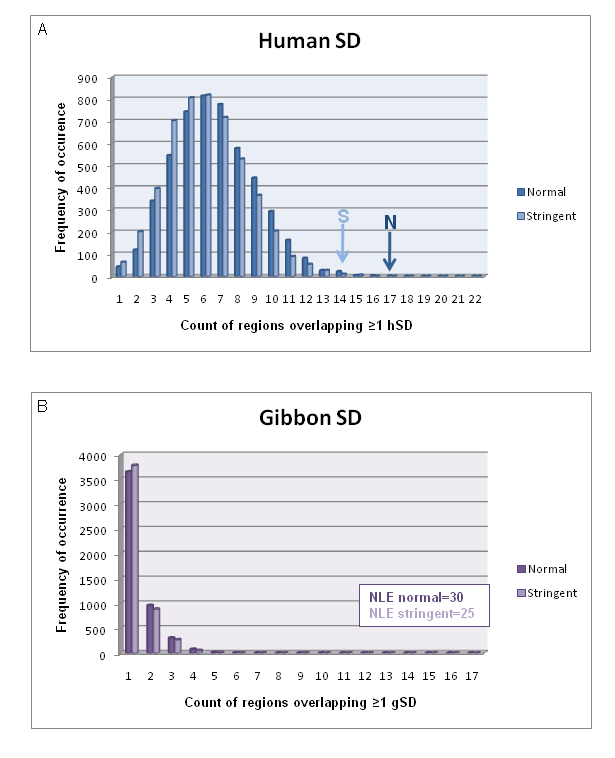

Supplement: Figure S1 — Random sampling simulations for human and gibbon segmental duplications. Random sampling simulations were carried on as described in Materials and Methods. Histograms were obtained for human SD (A) and the in silico set of gibbon SD (B). We also tested the overlap with a “stringent” sample (lighter color) where all the BP that in gibbon overlap with centromeres were removed. Even in this case it is evident that the overlap of the gibbon sample with both classes of SDs is significant. (1.42 MB TIF) [file pgen.1000538.s001.tif]

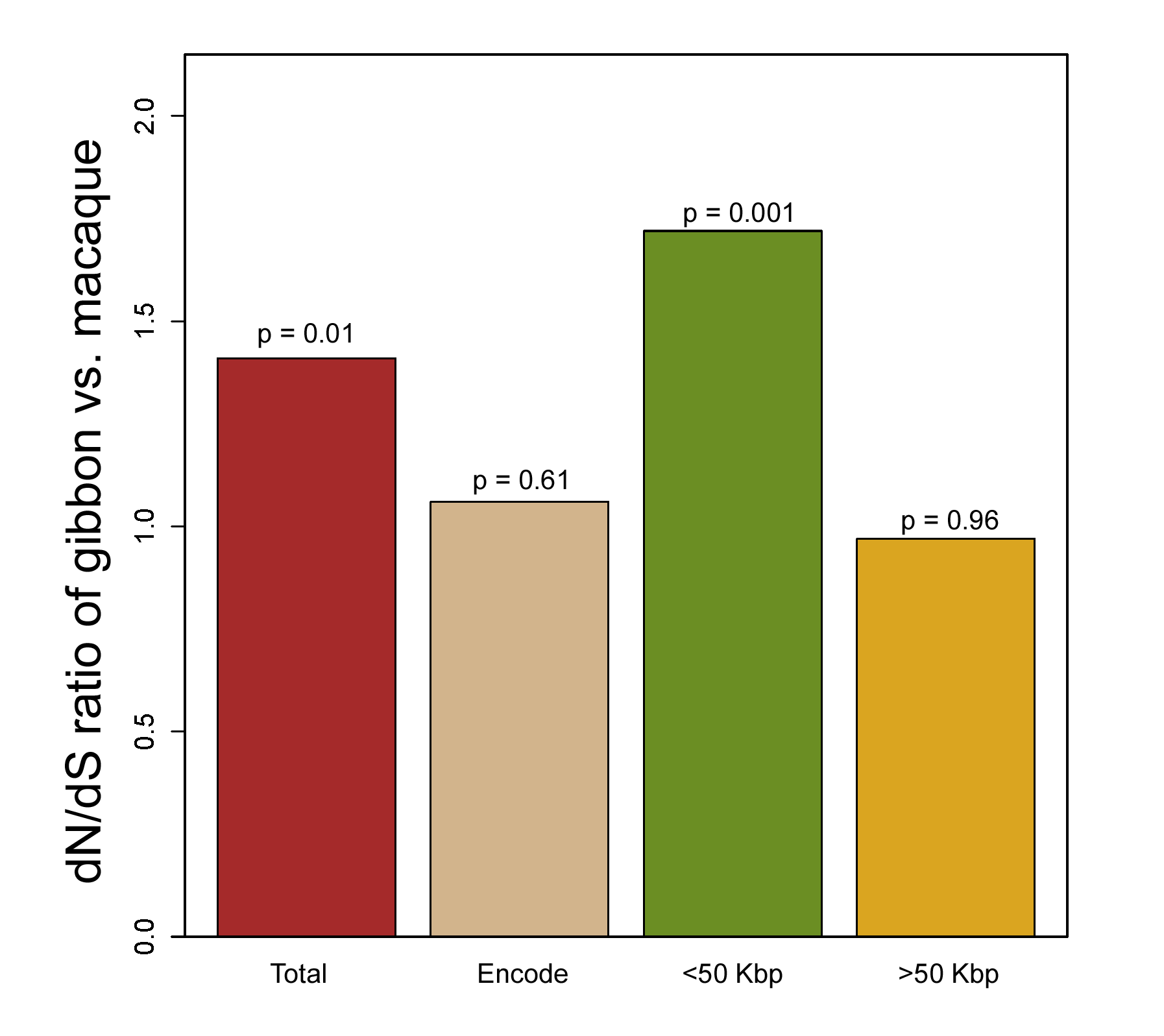

Supplement: Figure S2 — dN/dS ratios for gibbon and macaque genes. The ratio of the average dN/dS compute for gibbons (vs. human) and macaques (vs. human) for all genes found within the fully sequenced BACs (Total), for genes found within the NISC database (NISC), for genes located within 50 kb (<50 kb) from the breakpoint found within the BAC sequences and genes located further than 50 kb (>50 kb) from the breakpoint found within the BAC sequences. p values were calculated using the nonparametric Mann-Whitney test. (8.62 MB TIF) [file pgen.1000538.s002.tif]

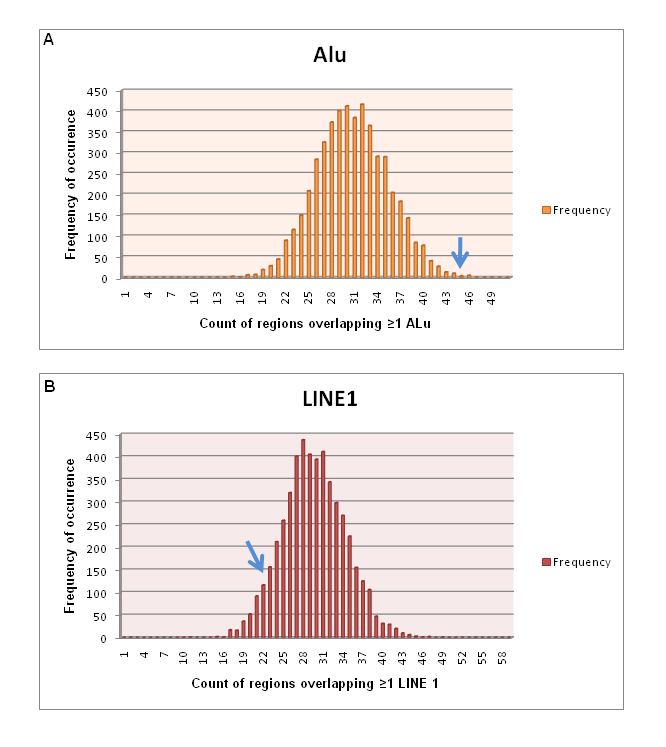

Supplement: Figure S3 — Random sampling simulations for Alu and Line 1 elements. Random sampling simulations were carried on as described in Materials and Methods. The two charts show the histogram resulted from counting the overlap between random regions of the human genome and Alu (A) and Line (B). The random sampling was repeated 5,000 times in both cases. The corresponding value for the gibbon dataset is indicated by the blue arrow. (1.51 MB TIF) [file pgen.1000538.s003.tif]
