# Supplementary material for: Evolutionary Breakpoints in the Gibbon Suggest Association between Cytosine Methylation and Karyotype Evolution
Source: PLoS Genet. 2009 Jun 26;5(6):e1000538. doi: 10.1371/journal.pgen.1000538 (PMC2695003; doi:10.1371/journal.pgen.1000538)
Supplement: Table S1 — Sequenced Gibbon BACs. The fully sequenced and assembled 23 gibbon BACs from the genomic BAC library CHORI-271 (http://bacpac.chori.org/library.php?id=228) are reported here with the corresponding accession numbers. (0.04 MB DOC) [file pgen.1000538.s004.doc]

| BAC | Acc Number |
| --- | --- |
| CH271-183B5 | CT954289 |
| CH271-224A19 | CT954290 |
| CH271-228C1 | CT954292 |
| CH271-228N13 | CT954293 |
| CH271-228P23 | CT954294 |
| CH271-246M2 | CT954296 |
| CH271-254H12 | CT954297 |
| CH271-261A22 | CT954298 |
| CH271-261K6 | CT954300 |
| CH271-261L1 | CT954301 |
| CH271-262E11 | CT954302 |
| CH271-263C9 | CT954303 |
| CH271-267G23 | CT954304 |
| CH271-275G5 | CT954309 |
| CH271-286K22 | CT954310 |
| CH271-372B11 | CT954312 |
| CH271-398E1 | CT954314 |
| CH271-78K20 | CT954320 |
| CH271-86M19 | CT954321 |
| CH271-236L11 | CT990551 |
| CH271-350B17 | CT990556 |
| CH271-446I8 | CT990557 |
| CH271-298N13 | CT990555 |
