# Supplementary material for: Evolutionary Breakpoints in the Gibbon Suggest Association between Cytosine Methylation and Karyotype Evolution
Source: PLoS Genet. 2009 Jun 26;5(6):e1000538. doi: 10.1371/journal.pgen.1000538 (PMC2695003; doi:10.1371/journal.pgen.1000538)
Supplement: Table S2 — Gibbon breakpoints mapping information and annotations. We report here the sequencing status and annotations for 80 BACs used in this study. All the BACs were End-sequenced and mapped on the human assembly (hg18). The breakpoint sequence was obtained for 46 BACs whereas for 7 clones we identify Trace Archives mate pairs whose mapping indicated the presence of the breakpoint but no breakpoint sequence was found. For 24 clones the breakpoints could not be narrowed down at a resolution higher than a BAC clone. For each breakpoint sequence repeat, segmental duplications and gene contents are annotated. Abbreviations: gSD = gibbon segmental duplication, hSD = human segmental duplication. (0.11 MB PDF) [file pgen.1000538.s005.pdf]

	BAC	BES1 and BES2 (hg18)	Rearrangement	BP name	BP position	Repeat	Segmental duplication	Genes (UCSC Ac.Nr or Ref.Seq)	Putative mechanism
Sequenced BACs	CH271-224A19	chr14:32617734-32618743	t(HSA14;HSA6)	224A19_BP_Left	chr14:32656636-32657143	none		NPAS3 (uc001wrs.1)	
		chr6:67272170-67272901		224A19_BP_Right	chr6:67444870-67445349		none		
	CH271-183B5	chr8:115974044-115974755	t(HSA8;HSA5)	183B5_BP1_Left	chr8:116206959-116207470	none			NHEJ (GA homology)
		_		183B5_BP1_Right	chr5:20662246-20662684		none		
		_	Inv(HSA5)	183B5_BP2_Left	chr5:20663011-20663381				
		chr5:20660946-20661339		183B5_BP2_Right	chr5:20661769-20662230	L1PA4/L1	none		
	CH271-228C1	chr7:79789663-79790600	t(HSA7;HSA20)	228C1_BP_left	chr7:79892914-79893600	(TA)n simple repeat (318bp)flanking		CD36 antigen (uc003uhc.1)	Stem-Loop fragility
		chr20:16651858-16651083		228C1_BP_right	chr20:16576945-16577328		none		
	CH271-228N13	chr10:56218669-56219382	Inv(HSA10)	228N13_BP_left	chr10:56188874-56189350		hSD at 11Kb from BP	PCDH15 (uc001jju.1)	
		chr10:88936450-88935739		228N13_BP_right	chr10:88788971-88789386	L1PA13/L1	hSD at 17Kb from BP		
	CH271-228P23	chr3:197132159-197133169	Inv(HSA3)	228P23_BP1_left	chr3:197102596-197103088	AluSp/q		TNK2 (uc003fvs.1)	
		_		228P23_BP1_right	chr3:196942308-196943209			MUC20 (NM_152673)	
		_	t(HSA3;HSA5)	228P23_BP2_left	chr3:196989840-196990455	Tandem-SVA-(GAAA)n		MUC4 (uc003fuz.1)	
		chr5:175546939-175548355		228P23_BP2_right	chr5:175653028-175653875	L1ME4a		C5orf25 (uc003mdr.1)	
	CH271-236L11	chr2:131700083-131701676	t(HSA2;HSA8)	236L11_BP1_Left	Ambiguous mapping	L1P/L1			
		_		236L11_BP1_Right	chr8:4398611-4399410	AluSx		CSMD1 (uc003wqd.1)	
		_	t(HSA8;HSA4)	236L11_BP2_Left	chr8:4361959-4362806	Block of repeats		CSMD1 (uc003wqd.1)	
		chr4:48733170-48734080		236L11_BP2_right	chr4:48761701-48762682				
	CH271-246M2	chr2:73408992-73409723	t(HSA17;HSA2)	246M2_BP_Left	chr2:73461817-73462374	LTR -AluJ-LTR			
		chr17 Ambiguous		246M2_BP_Right	Ambiguous mapping		hSD		
	CH271-254H12	chr5:54005950-54006867	t(HSA5;HSA16)	254H12_BP_Left	chr5:54116622-54116963	AluSx-(AT)n-BP-(AT)n		SULT1A1 (uc002dqm.1)	
		chr16:16100208-16100751		254H12_BP_Right	chr16:28539672-28540248		hSD and gSD		
	CH271-263C9	chr22:32009319-32010312	Inv(HSA22)	263C9_BP1_Left	chr22:31971622-31972151	Block of repeats	hSD		
		_		263C9_BP1_Right	chr22:30928393-30928889			RFPL2 (uc003ame.1)	
		_	t(HSA22;HSA4)	263C9_BP2_Left	chr22:31046644-31047107	AluSg		AK126764 (hypothetical)	NHEJ -gggtttcaggg-filling
		chr4:140563504-140564165		263C9_BP2_Right	chr4:140587411-140587918	C HERVL-int LTR (ERVL)			
	CH271-261L1	chr1:52150331-52151147	Inv(HSA1)	261L1_BP_Left	chr1:52327906-52328412	none	gSD		NHEJ "**aaggtg**" homology
		chr1:179414362-179415103		261L1_BP_Right	chr1:179425508-179425921				
	CH271-262E11	chr17:59260052-59260792	t(HSA17;HSA2)	262E11_BP_Left	chr17:59312447-59313017	AluS	gSD and hSD	GH2(uc002jcl.1); CSH1 (uc002jck.1)	Alu-Alu recombination (Figure 1)
		chr2:27718116-27718928		262E11_BP_Right	chr2:27780363-27780861				
	CH271-267G23	chr2:99339634-99340501**	t(HSA17;HSA2)	267G23_BP_Left	chr2:99288205-99289050	Block of repeats	gSD		
		chr17:77747544-77748535		267G23_BP_Right	chr17:77869060-77870607	(CG)n-simple repeat	gSD		
	CH271-78K20	chr4:117336722-117337842	t(HSA4;HSA16)	78K20_BP_Left	chr4:117280534-117281120		gSD		NHEJ -aattccaa-filling
		chr16:1603333216034231**		78K20_BP_Right	chr16:16101960-16102505	AluSg/x	gSD	ABCC1 (uc002dee.1)	
	CH271-86M19	chr7:80017020-80017854	t(HSA7;HSA20)	86M19_BP_Left	chr7:80017020-80017854	(AT)n-simple repeat		CD36 antigen (uc003uhc.1)	NHEJ -attccaagccatatattattgg-filling
		chr20:16553059-16553552		86M19_BP_Right	chr20:16553059-16553552		gSD		
	CH271-275G5	chr7:72639276-72639991	Inv(HSA7)	275G5_BP_Left	Ambiguous mapping			BAZ1B (uc003tyc.1)	
		chr7:99624194-99625196		275G5_BP_Right	Ambiguous mapping		hSD		
	CH271-350B17	chr4:117032787-117033832	t(HSA4;HSA16)	350B17_BP1_Left	chr4:117240577-117241153				NHEJ -ctccaacctt-filling
		_		350B17_BP1_Right	chr16:16111050-16111587	L2b/LINE2	gSD		
		_	Inv(HSA16)	350B17_BP2_Left	chr16:16118590-16119249	AluSx	gSD	ABCC1 (uc002dee.1)	NAHR between ABCC1-ABCC6
		_		350B17_BP2_Right	Ambiguous mapping	L1-AluSx-L1			
		_	t(HSA16;HSA5)	350B17_BP3_Left	Ambiguous mapping	L1M2/L1-AluSx-L1M2/L1			
		chr5:54236459-54237267		350B17_BP3_Right	chr5:54203270-54203913				
	CH271-398E1	chr17:69796721-69797532	Inv(HSA17)_1	398E1_BP1_Left	chr17:69836813-69837369	AluSc		KIF19 (uc002jkj.1)	
		_		398E1_BP1_Right	chr17:64467113-64467721	L1PA16/L1-THE1B/LTR		ABCA8 (uc002jhp.1)	
		_	Inv(HSA17)_2	398E1_BP2_Left	chr17:64442878-64443431	L1P5/LINE-L1M5/L1-1AluYd8-(CA)n-AluYd8-L1M5/L1		ABCA8 (uc002jhp.1)	Alu-Alu recombination (Figure1)
		chr17:20753342-20754118		398E1_BP2_Right	chr17:20786434-20787025				
	CH271-286K22	chr7:101923026-101923857	Inv(HSA7)	286K22_BP_Left	chr7:101978533-101978861	AluJo_1-AluSx_1-AluJo_1-AluSx_2_AluJo_1-AluSx_4	hSD		NAHR of hSD
		chr7:75864301-75865131		286K22_BP_Right	chr7:75987975-75988443		hSD	UPK3B (uc003ufq.1)	
	CH271-298N13 centromeric	chr18:14626725-14627692	t(HSA18;HSA2)	298N13_BP1_Left	chr18:14624361-14624802		hSD		
		_		298N13_BP1_Right	chr2:130451700-130452248	(CA)n-simple repeat -AluSc-AluJb	hSD		
		_	t(HSA2;HSA8)	298N13_BP2_Left	chr2:130315436-130316178	L1PA6/L1-L1ME3A/L1			
		_		298N13_BP2_Right	chr8:4317303-4317902		gSD		
		_	t(HSA8;HSA18)	298N13_BP3_Left	chr8:4260896-4261577		gSD	CSMD1 (uc003wqd.1)	NHEJ "**CT**" homology
		chr18:72428836-72429640		298N13_BP3_Right	chr18:72396578-72397187	AluJb	hSD		
	CH271-372B11 centromeric	chr14:19379603-19491075	t(HSA14;HSA2)	372B11_BP1_Left	Ambiguous mapping	L1M4c /L1-L1M1/L1-CL2/L2			
		_		372B11_BP1_Right	chr2:36968096-36968623		gSD		
		_	t(HSA2;HSA9)	372B11_BP2_Left	Ambiguous mapping	BP1-**AluJ/FRAM** -BP2-MLT1G3/LTR-L1MD/L1-**AluJo**			Alu-Alu recombination
		_		372B11_BP2_Right	chr9:98027851-98028324				
		_	t(HSA9;HSA6)	372B11_BP3_Left	chr9:97415689-97416270		gSD		NAHR of gSD
		chr6:151254410-151269850		372B11_BP3_Right	chr6:151263070-151263234		gSD		
	CH271-446I8 centromeric	chr7:100735913-100736921	Inv(HSA7)	446I8_BP_Left	chr7:100731021-100731650	AluSx -L1P3/1			Alu-Alu recombination
		chr7:68133693-68134788		446I8_BP_Right	chr7:68241607-68242220	L1ME/L1-AluSx-L1ME/L1			
	CH271-261K6 HSA specific	chr3:131140276-131141065	Inv(HSA3)	261K6_BP_Left	chr3:131200116-131200598	MIR (broken in human)	hSD on other side		NAHR of hSD in human
		chr3:15040075-15040867		261K6_BP_right	chr3:15138527-15139113	MIR-LTR16B2/LTR-MLT1D/LTR	gSD, hSD on other side		
	CH271-261A22 HSA specific	chr7:7101310-7102318	Inv(HSA7)	261A22_BP_Left	chr7:7006371-7006881	FLAM/SINE-LTR33/ERVL	hSD		NAHR of hSD in human
		chr7:97207081-97207691		261A22_BP_right	chr7:97340602-97341177		hSD		

	BAC	BES1 and BES2 (hg18)	Rearrangement	BP name	BP position	Repeat	Segmental duplication	Genes (UCSC Ac.Nr or Ref.Seq)	Putative mechanism
Shotgun Pool	CH271-125L9	chr3:147740472-147741241	t(HSA3;HSA12)	125L9_BP_Left	chr3:147732628-147732918	none	gSD	PLSCR1 (uc003evx.1)	
		chr12:45807865-45808660		125L9_BP_Right	chr12:45890669-45891125			FAM113B (uc001rpn.1)	
	CH271-141K21	chr9:113404746-113404882	Inv(HSA9)	141K21_BP_Left	chr9:113460127-113460458			LOC552891 (uc004bfn.1)	
		chr9:22218708-22218920		141K21_BP_Right	chr9:22288734-22289442	L1M1/L1			
	CH271-185K6	chr14:30838138-30838392	Inv(HSA14)	185K6_BP_Left	chr14:30985515-31679560*	AluY			
		chr14:73124155-73124877		185K6_BP_Right	chr14:73091554-73091829	L1M1/L1		HEATR4 (uc001xoj.1)	
	CH271-18C15	chr6:9423952-9424795	t(HSA6;HSA11)	18C15_BP_Left	chr6:9375117-9375652	none	gSD		
		chr11:32321052-32321338		18C15_BP_Right	chr11:32145365-32145812	none	gSD		
	CH271-241J10	chr1:54907463-54907795	Inv(HSA1)	241J10_BP_Left	chr1:55009636-55010105	L2a/L2	gSD		NHEJ -AAAAAAAAAATTTTCT-filling
		chr1:211021684-211022133		241J10_BP_Right	chr1:211097036-211097354				
	CH271-274L1	chr17:69720630-69721057	Inv(HSA17)_1	274L1_BP1_Left	chr17:69833458-69833569				NHEJ -TGGTATGGAGCGAGCACCTCA-filling
		_		274L1_BP1_Right	chr17:70243897-70244356	Alu		RAB37 (uc002jle.1)	
		_	Inv(HSA17)_2	274L1_BP2_Left	No Breakpoint Information				
		chr17:66671503-66672271		274L1_BP2_Right					
	CH271-330D2	chr16:73591531-73592258	t(HSA16;HSA5)	330D2_BP_Left	chr16:73411618-73411924	L1			NHEJ "**CA**" homology
		chr5:132444217-132444431		330D2_BP_Right	chr5:132470668-132471176	AluJb at 210bp			
	CH271-362G10	chr6:27185623-27186149	t(HSA6;HSA9)	362G10_BP_Left	chr6:27133029-27133530		gSD		
		chr9:30805802-30806750		362G10_BP_Right	chr9:30938218-30938483	L1MEc/L1			
	CH271-405A9	chr2:27994865-27994148	t(HSA2;HSA17)_RecCH271-262E11	405A9_BP_Left	chr2:27780864-27781537	MIR3-AluSg/x			
		chr17:59333868-59333191		405A9_BP_Right	chr17:59313018-59313274		gSD and hSD		
	CH271-44A23	chr7:2700908-2701376	t(HSA7;HSA2)	44A23_BP_Left	chr7:2619384-2620096		gSD		
		chr2:150152851-150153542		44A23_BP_Right	chr2:150079919-150080449	AluS			
	CH271-171B20	chr9:32478670-32479624	Inv(HSA9)_1	171B20_BP1_Left	chr9:32478468-32478924	AluSx			NHEJ -ATACTACATATATAGA(TA)$_5$TCCT-filling
		_		171B20_BP1_Right	chr9:38261234-38261674				
		_	Inv(HSA9)_2	171B20_BP2_Left	chr9:38779194-38779598	No Breakpoint sequence			
		chr9:27166326-27166532		171B20_BP2_Right	chr9:27256349-27256935				
	CH271-185E17	chr8:62776249-62776628	Inv(HSA8)	185E17_BP_Left	chr8:62850735-62850926				
		chr8:99254477-99255380		185E17_BP_Right	chr8:99136628-99137178	AluSg/x			
	CH271-122E24	chr6:46092903-46093778	t(HSA6;HSA2)	122E24_BP_Left	chr6:46224094-46224375	AluY-(TTTA)n -AluY			
		chr2:168833875-168834096		122E24_BP_Right	chr2:168921485-168921828				
	CH271-40A18	chr6:46243705-46246528	t(HSA6;HSA2)_RecCH271-122E24	40A18_BP_Left	chr6:46244450-46244952	(TA)n			
		chr2:168943139-168960712		40A18_BP_Right	chr2:168941518-168941810	L1			
	CH271-449L10	chr12:63651232-63652094	t(HSA12;HSA19)	449L1_BP_Left	chr12:63567426-63567984	AluJo			NHEJ-AAAA-filling
		chr19:41725837-41726391		449L1_BP_Right	chr19:41824636-41824883				
	CH271-234B14	chr3:19775959-19776713	t(HSA3;HSA8)	234B14_BP_Left	chr3:19801280-19801846	Tigger3b DNA/MER2			
		chr8:20169960-20170244		234B14_BP_Right	chr8:19972153-19972405	THE1A/LTR-L1MA3/L1			
	CH271-49C12	chr17:55502427-55503122	Inv(HSA17)_1	49C12_BP1_Left	chr17:55479737-55479892			ABC1 (uc002iyk.1)	
		_		49C12_BP1_Right	chr17:27828000-27828705			PSMD11 (uc002hhm.1)	
		_	Inv(HSA17)_2	49C12_BP2_Left	Missing	AluY			
		chr17:45884382-45884620		49C12_BP2_Right	Missing				
	CH271-107F17	chr22:32050317-32050909	Inv(HSA22)	107F17_BP_Left	chr22:31971471-31971840	HSMAR2/Mariner-AluSx- HSMAR2/Mariner			
		chr22:31006873-31007483		107F17_BP_Right	chr22:30920732-30920843		hSD	RFPL2 (uc003ame.1)	
	CH271-114O8	chr5:75772933-75773648	t(HSA5;HSA16)	114O8_BP_Left	chr5:75703809-75704511				NHEJ-ATGATG-filling
		chr16:19525951-19526628		114O8_BP_Right	chr16:19419664-19419762				
	CH271-247C2	chr2:99193372-99193548	t(HSA2;HSA17)_RecCH271-267G23	247C2_BP_Left	chr2:99287211-99287966	AluY			
		chr17:77943112-77943299		247C2_BP_Right	chr17:77869210-77870732				
	CH271-438C12	chr10:52579011-52579213	t(HSA10;HSA14)	438C12_BP_Left	chr10:52595969-52596550	MSTA/LTR		PRKG1 (uc001jjm.1)	NHEJ-AAC-filling
		chr14:35365902-35366597		438C12_BP_Right	chr14:106265726-106265962			abParts (uc001yrq.1)	
	CH271-383H22	chr3:194767531-194767746	Inv(HSA3)_1	383H22_BP1_Left	chr3:194750831-194751067	AluJb	gSD		Alu-Alu recombination
		_		383H22_BP1_Right	chr3:198930706-198931429	L1MA6/L1			
		_	Inv(HSA3)_2	383H22_BP2_Left	Missing				
		chr3:196990112-196990361		383H22_BP2_Right	Missing				
Trace Archives	Trace:1744822164	_	Inv(HSA17)_RecCH271-389E1	Rec_398E1_BP_Left	chr17:64468378-64468996	Charlie			NHEJ-GAAATAGAAATAAAAAC-filling
		_		Rec_398E1_BP_Left	chr17:69838399-69838549			KIF19 (uc002jkj.1)	
	CH271-119L23	chr19:44062149-44062967	t(HSA19;HSA7)	119L13_BP_Left	chr19:44009702-44014702	**No Breakpoint sequence**		HNRNPL (uc002ojk.1); ECH1 (uc002oji.1)	**No Breakpoint sequence**
		chr7:23148716-23149425		119L13_BP_Right	chr7:23062779-23067779				
	CH271-262E15	chr4:187202780-187203261	Inv(HSA4)	262E15_BP_Left	chr4:187202337-187207337				
		chr4:185313479-185314230		262E15_BP_Right	chr4:185309892-185314892			ENPP6 (uc003iwc.1)	
	CH271-401L9	chr10:24076626-24076771	t(HSA10;HSA4)	401L9_BP_Left	chr10:23994393-23999393				
		chr4:110612050-110612158		401L9_BP_Right	chr4:110501558-110506558				
	CH271-457L13	chr22:31172669-31173346	t(HSA22;HSA4)_RecCH271-263C9	457L13_BP_Left	chr22:31042892-31047892		hSD		
		chr4:140618444-140619353		457L13_BP_Right	chr4:140586473-140591473		gSD		
	CH271-300N11	chr10:11957906-11958581	Inv(HSA10)	300N11_BP_Left	chr10:12128225-12133225				
		chr10:15049040-15049323		300N11_BP_Right	chr10:15063576-15068576		hSD		
	CH271-274K2	chr2:128217917-128218714	Inv(HSA2)	274K2_BP_Left	chr2:128110049-128115049			LIMS2 (uc002tou.1)	
		chr2:131832424-131832748		274K2_BP_Right	chr2:131905153-131910153				
	CH271-322A3	chr13:38382334-38382944	Inv(HSA13)	322A3_BP_Left	chr13:38336699-38341699			FREM2 (uc001uwv.1)	
		chr13:22312091-22312355		322A3_BP_Right	chr13:23436399-23441399				

	BAC	BES1 and BES2 (hg18)	Rearrangement	BP name	BP position	Repeat	Segmental duplication	Genes (UCSC Ac.Nr or Ref.Seq)	Putative mechanism
BES Only	CH271-212J20	chr15:28990033-28990789<br>chr15:30748924-30749757	Inv(HSA15)	No Breakpoint mapping Information					
	CH271-219C13/CH271-156E13	chr2:73614518-73615293<br>chr17:61651667-61651884	t(HSA2;HSA17)						
	CH271-219C17	chr17:20464920-20494920<br>chr17:57601971-57631971	Inv(HSA17)						
	CH271-186A1	chr6:57494796-57495539<br>chr6:62082461-62083238	Inv(HSA6)						
	CH271-144D7	chr17:33747350-33748174<br>chr17:43053145-43053947	Inv(HSA17)						
	CH271-144P05	chr17:43030034-43030452<br>chr17:45903313-45904103	Inv(HSA17)						
	CH271-244C18	chr6:26665529-26666210<br>chr6:58278082-58278396	Inv(HSA6)						
	CH271-133C19	chr17:27546813-27547000<br>chr17:26246352-26246485	Inv(HSA17)						
	CH271-362O01	chr2:114170325-114170537<br>chr2:132955805-132956199	Inv(HSA2)						
	CH271-314F10	chr18:10502587-10502787<br>chr18:12104383-12105287	Inv(HSA18)						
	CH271-10M04	chr11:3137758-3138457<br>chr11:70938138-70938965	Inv(HSA11)						
	CH271-316D01	chr12:50750486-50750968<br>chr3:76165224-76165428	t(HSA12;HSA3)						
	CH271-380N5	chr3:58787556-58788437<br>chr12:120366540-120367347	t(HSA12;HSA3)						
	CH271-461L14	chr13:26172852-26173025<br>chr1:202036523-202037082	t(HSA13;HSA1)						
	CH271-202N11	chr10:51751461-51752217<br>chr10:52229449-52229635	Inv(HSA10)						
	CH271-263M06	chr7:102292933-102293699<br>chr7:92995033-92995531	Inv(HSA7)						
	CH271-267G21	chr17:55284622-55285294<br>chr17:57642889-57643214	Inv(HSA17)						
	CH271-26L7	chr17:45966195-45967178<br>chr17:30504099-30504910	Inv(HSA17)						
	CH271-134D13	chrX:34418293-34418679<br>chrX:63295824-63296135	Inv(HSAX)						
	CH271-64D21	chrX:36155073-36155711<br>chrX:34207559-34208240	Inv(HSAX)						
	CH271-262O07	chr8:116011454-116012115<br>chr5:20660956-20661663	t(HSA8;HSA5)						
	CH271-30L2	chr19:5851377-5851846<br>chr19:51500098-51500471	Inv(HSA19)						
	CH271-74B2	chr17:61598754-61599496<br>chr17:20111039-20111836	Inv(HSA17)						
	CH271-216H2	chr10:52022220-52023160<br>chr10:89294947-89295755	Inv(HSA10)						
False Positives	CH271-16A10	No Breakpoints							
	CH271-146B14								
	CH271-188I7								
