# Supplementary material for: Evolutionary Breakpoints in the Gibbon Suggest Association between Cytosine Methylation and Karyotype Evolution
Source: PLoS Genet. 2009 Jun 26;5(6):e1000538. doi: 10.1371/journal.pgen.1000538 (PMC2695003; doi:10.1371/journal.pgen.1000538)
Supplement: Protocol S1 — Breakpoints mapping strategy. (0.03 MB DOC) [file pgen.1000538.s009.doc]

**Protocol S1**

**Breakpoints mapping**

The 57 BAC clones were then divided into 2 pools with the purpose of keeping apart clones whose BES map in very close regions on the human genome and whose mapping would create ambiguity. Single BAC cultures were set up in order to avoid biases due to variable speed of growth. After overnight growth the DNA was extracted using the automated system AUTOGEN 960 and then combined to create 2 pools. Each DNA pool was subsequently purified using CsCl gradient to eliminate any chromosomal DNA contaminations. A shotgun library was created from each pool using standard protocols. Briefly, the DNA was shared using Hydroshare apparatus and end-repaired. Sizes between 2 and 4 Kbp were gel-selected, DNA was recovered from the gel by electroelution and dialyzed against TE. The insert DNA was then ligated to the pUC19 liberalized vector and electroporated into DH10B cells (Invitrogen). The insert ends of the shotgun clones were sequenced using standard primers (M13 and t7) and mapped on the human genome by BLAT. A pairing script was used to identify breakpoints of translocations or inversions and pairing clones. In few instances the breakpoint fell inside a sequenced end of a shotgun clone or in the un-sequenced portion of it allowing us to easily retrieve the gibbon specific sequence at the breakpoint site. In all the other cases we used the indicative position of the breakpoint to retrieve the gibbon sequences from the trace archives by using the sequence of the human region as a probe to BLAST against the *Nomascus leucogenys leucogenys* trace archives.
