# Supplementary material for: Evolutionary Breakpoints in the Gibbon Suggest Association between Cytosine Methylation and Karyotype Evolution
Source: PLoS Genet. 2009 Jun 26;5(6):e1000538. doi: 10.1371/journal.pgen.1000538 (PMC2695003; doi:10.1371/journal.pgen.1000538)
Supplement: Protocol S2 — dN/dS analysis. (0.03 MB DOC) [file pgen.1000538.s010.doc]

**Protocol S2**

**dN/dS analysis**

We identified 72 (Supplemental Table 1) genes with sequences readily available for humans, gibbons and macaques from the NIH Intramural Sequencing Center archive (www.nisc.nih.gov). For regions where macaque sequences were not available, we used the coordinates of the genes to extract the macaque reference sequence (Jan.2006/rheMac2 build) from the UCSC genome browser (www.genome.ucsc.edu). We then identified 49 (Supplemental Table 2) additional genes after partitioning the BAC sequence into 20 Kbp reads and locating regions where all three sequences were known. All human coordinates and reference sequences are based on NCBI build 36 of the human genome. We did not include not coding RNAs in this analysis.

Three-way sequence alignments were analyzed by PAML1 [43] (Phylogenetic Analysis by Maximum Likelihood). dS and dN was estimated for each coding sequence by PAML's subroutine codeml with the model assumption of one dN/dS ratio for all lineages. As different isoforms of the same gene may provide different estimates of dS and dN, we present the minimum dN/dS ratio for each particular gene. To determine significance of dN/dS ratio estimated for gibbons and macaques, we conduct the nonparametric Mann-Whitney U test. We found non-significant differences for genes found in the NISC database (p=0.605) but found significant differences for genes found near the breakpoints in the BAC sequences (p=0.002) (Supplemental Table 3). Furthermore, after removal of 4 genes for insufficient alignment data, 4 hypothetical genes, and 6 genes found in BAC 262E11, we still maintain significant differences for genes found in the BAC sequences (p=0.01). The 6 genes in the left arm of BAC 262e11 were omitted because they belong to a gene cluster family with high sequence identity. This sequence identity along with their positions upstream and downstream of the breakpoint lead to uncertainty in alignment data and gene coordinates. We next evaluated differences in dN/dS ratio for genes found within 50kb of the break point and genes found further than 50 Kbp. Distances were determined by calculating the minimum distance from either the start or end of the gene to the breakpoint. We find significant differences for genes found within 50 Kbp of the breakpoint (p=0.001) and non-significant differences for genes found further than 50 Kbp of the breakpoint (p=0.96) (Supplemental Table 4).

We explored whether or not early termination would impact our findings. We define genes with early terminations as those where we have alignment sequences for the entire gene and if either the gibbon or macaque sequence contained a stop codon other than (and earlier than) the proposed human stop site. We identify 3 out of 44 NISC genes and 4 out of 19 BAC genes which possibly contains an early termination site. Fisher's exact test yields non-significant results (p=0.355). Since the distribution of early terminations are not significant, this suggests that the positional effects observed are not due to early termination sites.
